# Standardized Hydroxytyrosol-Enriched Olive Pomace Juice Modulates Metabolic and Neurotrophic Signaling Pathways to Attenuate Neuroinflammation and Protect Neuronal Cells

**DOI:** 10.3390/molecules31020336

**Published:** 2026-01-19

**Authors:** Ye-Lim You, Ha-Jun Byun, Namgil Kang, Min Soo Lee, Jeong-In Lee, Ilbum Park, Hyeon-Son Choi

**Affiliations:** 1 Department of Food Nutrition, Sangmyung University, Hongjimun 2-gil 20, Jongno-gu, Seoul 03016, Republic of Korea; 202333023@sangmyung.kr (Y.-L.Y.); byunhajun@gmail.com (H.-J.B.); 2 JY Globalfoods Co., Ltd., #304, 1-Dong, SKV1 Center, 48, Achasan-ro 17-gil, Seongdong-gu, Seoul 04799, Republic of Korea; team17@unibio.kr (N.K.); lms@jyglobalfoods.com (M.S.L.);; 3 Kwangdong Pharm Co., Ltd., 52, Gwacheon-daero 7da-gil, Gwacheon-si 13840, Republic of Korea; pib975@ekdp.com

**Keywords:** olive pomace juice (OPJ), hydroxytyrosol, neuroprotection, neuroinflammation, Nrf2, AMPK, CREB

## Abstract

Olive pomace (OP), a by-product of olive oil production, is a sustainable resource rich in bioactive compounds with potential applications in cosmetics and pharmaceuticals. This study investigates the protective effects of olive pomace juice (OPJ) against H_2_O_2_-induced neuronal damage and LPS-induced inflammatory responses in HT22 and BV2 cells, respectively. OPJ suppressed H_2_O_2_-induced cell death and exerted anti-apoptotic effects by reducing the BAX/BCL2 ratio and caspase-3 cleavage. OPJ also mitigated neurodegenerative hallmarks by decreasing amyloid fibrils formation and inhibiting β-secretase and acetylcholinesterase (AChE) activity. Mechanistically, OPJ enhanced antioxidant response by upregulating Nrf2 and its downstream molecule HO-1, along with increasing mRNA levels of antioxidant enzymes, including catalase, SOD1, and GPx. OPJ further activated AMPKα–SIRT1–PGC1α signaling and CREB–BDNF–TrkB signaling, suggesting modulation of key antioxidant, anti-apoptotic, and neurotrophic pathways. In BV2 cells, OPJ downregulated pro-inflammatory cytokines (IL-6 and IL-1β) and decreased iNOS and COX-2 expression through suppression of NF-κB and MAPK signaling pathways. HPLC analysis identified hydroxytyrosol (10.92%) as the major active compound in OPJ, which compared with tyrosol (2.18%), and hydroxytyrosol exhibited greater neuroprotective and anti-inflammatory effects than tyrosol. This study highlights the potential of OPJ and its major compound, hydroxytyrosol, as functional agents for mitigating neurodegeneration-related cellular response, supporting its application in the food and pharmaceutical industries.

## 1. Introduction

The olive tree (*Olea europaea* L.) and its fruit have been highly valued for centuries for their culinary significance and diverse applications in food, health, and cosmetics [1,2]. Olives are prized for their rich flavor, nutritional value, and versatility, packed with bioactive compounds primarily found in olive oil, olive leaves, and the fruit itself [3]. These compounds offer a wide range of health benefits, including antioxidant and anti-inflammatory effects, and contribute to cardiovascular health, skin rejuvenation, and brain function [4,5]. Beyond these applications, olive derivatives also play an important role in industrial processes, including biofuel production and animal feed, highlighting their remarkable versatility [6,7]. One by-product of olive oil production, olive pomace, consists of the solid residue left after oil extraction, including skins, pulp, seeds, and stems [2]. Traditionally considered waste, olive pomace is now gaining attention due to its nutritional value, bioactive compounds, and functional properties, opening up potential applications across various industries [1]. In the broader context of sustainability, the food industry—despite its wide-ranging applications—remains one of the largest contributors to global waste, creating significant environmental, social, and economic challenges [8]. Addressing these challenges requires effective waste management strategies that emphasize resource efficiency. Upcycling, which transforms by-products into higher value goods, is a promising approach [9], making the olive industry an excellent example of contributing to a more sustainable food system [10].

Neurodegenerative diseases are global health challenges of growing concern, standing out due to their widespread impact and increasing prevalence [11,12]. These conditions such as Alzheimer’s, Parkinson’s diseases, are marked by the accumulation of toxic proteins, oxidative stress, inflammation, and mitochondrial dysfunction [13,14,15]. The rising incidence is largely due to the aging population, the most significant risk factor [16]. While current pharmacological treatments can help slow symptom progression, they are often associated with side effects such as nausea, diarrhea, and loss of appetite [5], leading to a growing demand for natural products or food-based therapies that offer long-term benefits with minimal side effects.

Recent research has highlighted the neuroprotective potential of olives and their bioactive compounds against the molecular and cellular mechanisms that underlie neurodegenerative diseases [1,5,17,18,19]. However, although the neuroprotective effects of major olive polyphenols have been widely reported, these effects have not been investigated in an integrated manner that includes the AMP-activated protein kinase alpha (AMPKα)–sirtuin 1 (SIRT1)–peroxisome proliferator-activated receptor gamma coactivator 1 alpha (PGC1α) signaling axis. Notably, the AMPKα–SIRT1–PGC1α signaling axis promotes mitochondrial biogenesis and enhances antioxidant defenses, thereby countering oxidative stress, a major contributor to neuronal degeneration [20]. AMPKα represents the catalytically active α-subunit of AMP-activated protein kinase and serves as a central regulator of cellular energy sensing and redox homeostasis. Similarly, PGC1α is a major α-isoform-specific transcriptional coactivator that governs mitochondrial biogenesis and oxidative metabolism downstream of AMPK signaling. These molecules were therefore selected as key signaling targets to evaluate OPJ-mediated regulation of energy homeostasis and redox balance in neuronal cells. Similarly, the cAMP response element-binding protein (CREB)–brain-derived neurotrophic factor (BDNF)–tropomyosin receptor kinase B (TrkB) pathway plays a crucial role in neuronal survival and synaptic plasticity, providing resilience against progressive neuronal loss [8]. Despite their relevance, involvement of these pathways in neuroprotective effects of olive pomace has not yet been explored, representing a key mechanistic gap that this study aims to address.

Although the neuroprotective effects of individual olive polyphenols, such as hydroxytyrosol, tyrosol, and oleuropein, have been extensively reported—primarily in relation to antioxidant and anti-inflammatory mechanisms—these studies have largely focused on isolated compounds and single signaling pathways. Consequently, whether olive pomace juice, as a complex polyphenolic matrix, is associated with integrated modulation of multiple stress-responsive signaling networks relevant to neuronal resilience remains unclear.

Based on this mechanistic gap, the present study tests the hypothesis that olive pomace juice is associated with changes in key signaling pathways involved in oxidative stress regulation, metabolic homeostasis, neurotrophic support, and inflammatory responses, including the AMPKα–SIRT1–PGC1α, CREB–BDNF–TrkB, and NF-κB pathways, in mouse hippocampal neuronal (HT22) and microglial (BV2) cell models. By focusing on extract-level associations rather than attributing effects to individual compounds, this study provides novel integrative insight into how olive pomace-derived polyphenols may collectively influence neuronal stress responses. In addition, the findings support the potential value of olive pomace as a sustainable, food-derived resource for neuroprotective research.

## 2. Results and Discussion

### 2.1. High-Performance Liquid Chromatography (HPLC) Analysis of Hydroxytyrosol and Tyrosol in OPJ

To characterize the major phenolic constituents of olive pomace juice (OPJ), hydroxytyrosol and tyrosol were quantified using high-performance liquid chromatography (HPLC). Representative HPLC chromatograms of OPJ showed well-resolved peaks corresponding to hydroxytyrosol and tyrosol, which were identified by comparison with authentic standards based on retention times and UV spectral characteristics (Figure S1A–C). Quantitative analysis revealed that hydroxytyrosol was the predominant phenolic constituent in OPJ, accounting for 10.92% of the total detected compounds, whereas tyrosol was present at a substantially lower proportion (2.18%). In addition, a considerable fraction of the total polyphenol content (21.46%), as determined by the Folin–Ciocalteu colorimetric assay, consisted of unidentified polyphenolic compounds. Notably, oleuropein, which is commonly reported as a major phenolic component in olive-derived products, was not observed under the specific HPLC conditions employed in this study, which were optimized for the targeted quantification of hydroxytyrosol and tyrosol rather than comprehensive phenolic profiling. Based on their relative abundance among the quantified phenolic compounds, hydroxytyrosol and tyrosol were selected as representative phenolic constituents for subsequent cellular experiments. The concentrations of OPJ used in subsequent cell-based experiments were expressed as μg/mL of the OPJ extract, reflecting the total extract mass containing the quantified phenolic constituents, rather than the concentrations of individual polyphenols.

**Figure 1:**
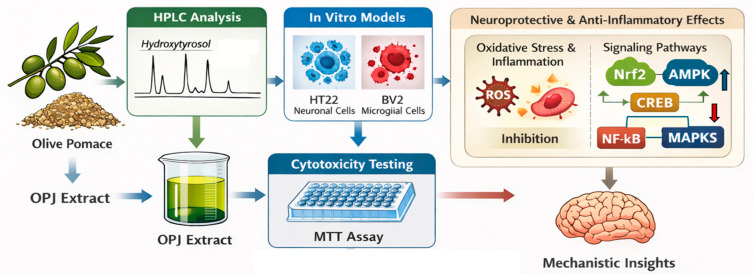
Experimental workflow for olive pomace juice (OPJ) study.

An overview of the experimental workflow is summarized in Figure 1.

### 2.2. OPJ Protects HT22 Neurons Against H_2_O_2_-Induced Oxidative Damage

To evaluate whether olive pomace juice (OPJ) confers direct neuroprotective effects against oxidative stress-induced neuronal injury, HT22 hippocampal neuronal cells were used as an in vitro model. Cell viability was assessed using an MTT assay under hydrogen peroxide (H_2_O_2_)-induced oxidative stress conditions. In this study, the normal untreated group without H_2_O_2_ exposure was defined as the normal control group (NOR), while cells treated with H_2_O_2_ alone served as the oxidative stress control group (CON).

**Figure 2:**
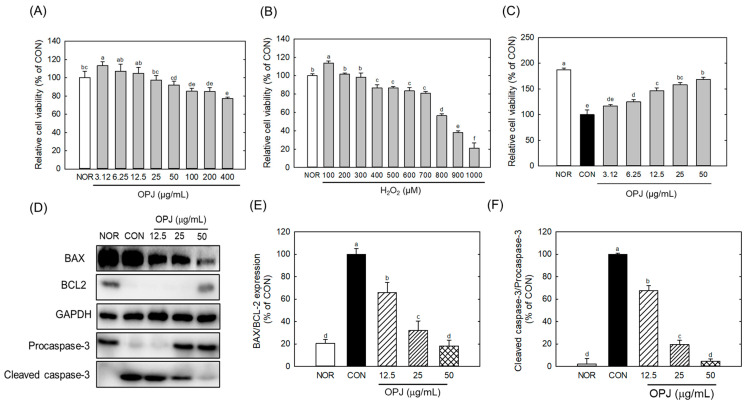
Effect of OPJ on viability and anti-apoptotic signaling in HT22 cells exposed to oxidative stress. HT22 hippocampal neuronal cells were incubated for 24 h with OPJ (3.125–400 μg/mL) (**A**), hydrogen peroxide (H_2_O_2_; 100–1000 μM) alone (**B**), or OPJ under H_2_O_2_-induced oxidative stress (800 μM) (**C**), and cell viability was assessed using an MTT assay. Protein abundances of BAX, BCL2, total caspase-3, cleaved caspase-3, and GAPDH were determined by Western blot analysis (**D**–**F**). Relative protein levels of BAX/BCL2 (**E**) and cleaved caspase-3/total caspase-3 (**F**) were quantified using ImageJ (Version 1.53t). All values represent mean ± SEM. Means with different letters indicate significant differences (*p* < 0.05). NOR, normal untreated group; CON, H_2_O_2_-treated control group (800 μM).

The cytotoxicity of OPJ was first evaluated in HT22 neuronal cells under basal conditions without H_2_O_2_-induced oxidative stress to determine appropriate treatment concentrations. OPJ slightly increased HT22 cell viability at low concentrations ranging from 3.12 to 12.5 μg/mL. OPJ treatment at 25 and 50 μg/mL did not significantly affect cell viability compared with the control (CON) group. In contrast, OPJ at concentrations of 100 μg/mL and above significantly reduced HT22 cell viability in a dose-dependent manner, with a 23% reduction observed at 400 μg/mL (Figure 2A). Based on these results, non-cytotoxic concentrations of OPJ (12.5, 25, and 50 μg/mL) were selected for all subsequent experiments.

Hydrogen peroxide (H_2_O_2_) treatment induced a dose-dependent reduction in HT22 cell viability. Notably, exposure to 800 μM H_2_O_2_ decreased cell viability by 44.6% relative to the CON group, indicating substantial oxidative cytotoxicity (Figure 2B). Under oxidative stress conditions induced by 800 μM H_2_O_2_, OPJ treatment significantly enhanced HT22 cell viability in a dose-dependent manner. OPJ concentrations up to 50 μg/mL increased cell viability by approximately 68% compared with the H_2_O_2_-treated group (Figure 2C), demonstrating a pronounced neuroprotective effect against oxidative injury.

Sustained oxidative stress induced by H_2_O_2_, a potent reactive oxygen species (ROS) generator, is a major contributor to neuronal injury through disruption of cellular redox homeostasis and mitochondrial dysfunction. In HT22 cells, the marked reduction in cell viability following H_2_O_2_ exposure reflects neuronal vulnerability to oxidative insults, which are closely associated with early molecular events in neurodegenerative processes, including dysregulation of β-secretase (BACE1) and acetylcholinesterase (AChE). The restoration of cell viability by OPJ under oxidative stress conditions suggests that OPJ effectively mitigates ROS-induced neuronal damage and preserves cellular integrity.

These findings establish HT22 neurons as a primary model for evaluating the neuroprotective effects of OPJ and provide a foundation for subsequent analyses of apoptosis regulation and neurodegeneration-related biomarkers described in the following sections.

### 2.3. OPJ Inhibits Oxidative Stress-Induced Apoptosis by Modulating BAX/BCL2 Signaling in HT22 Cells

To determine whether the neuroprotective effects of OPJ against oxidative stress involve regulation of apoptotic signaling, the expression of key apoptosis-related proteins was analyzed in H_2_O_2_-exposed HT22 neuronal cells.

Western blot analysis revealed that H_2_O_2_-induced oxidative stress markedly altered the expression of multiple apoptosis-related proteins, including BAX, BCL2, and caspase-3, in HT22 neuronal cells (Figure 2D). Specifically, H_2_O_2_ treatment significantly reduced the levels of BCL2, an anti-apoptotic factor. In contrast, OPJ treatment markedly increased its protein abundance at concentrations of 50 μg/mL, compared to the control group (CON) (Figure 2D). OPJ demonstrated an anti-apoptotic effect by reducing the ratio of Bcl-2–associated X protein (BAX) to B-cell lymphoma 2 (BCL2), an indicator of apoptosis, by 34.4%, 68.2%, and 81.8% at concentrations of 12.5, 25, and 50 μg/mL, respectively (Figure 2E). Additionally, caspase-3, a terminal effector of apoptosis, was significantly regulated by OPJ treatment. The inactive form of caspase-3 (procaspase-3) increased with OPJ treatment, while the activated form (cleaved caspase-3) decreased. OPJ reduced the ratio of cleaved caspase-3/caspase-3, another indicator of apoptosis, by 32.1%, 80.4%, and 95.3% at 12.5, 25, and 50 μg/mL, respectively (Figure 2F). These results suggest that OPJ effectively protects neuronal cells from H_2_O_2_-induced damage by modulating apoptosis-related proteins and pathways. Hydrogen peroxide (H_2_O_2_)-induced oxidative stress is a well-established trigger of neuronal apoptosis, in which ROS-mediated damage to proteins, lipids, and DNA disrupts cellular integrity [21]. In neurons, such oxidative insults shift the balance toward pro-apoptotic signaling, creating an environment that favors programmed cell death [21]. Our findings indicate that OPJ counteracts these oxidative stress-driven mechanisms, exerting neuroprotective effects by modulating the apoptotic machinery rather than merely preventing ROS accumulation. Consistent with these observations, OPJ attenuated the increase of pro-apoptotic mediators, including BAX induction and caspase-3 cleavage, while enhancing the stability of anti-apoptotic proteins such as BCL2 and procaspase-3. This modulation restores the balance between survival and death pathways, suggesting that OPJ not only protects structural integrity but also actively reprograms the apoptotic threshold in neuronal cells. Beyond the regulation of apoptosis-related proteins, OPJ may help preserve mitochondrial function and maintain cellular energy homeostasis under oxidative stress conditions [22,23]. By recalibrating the BAX/BCL2 ratio and inhibiting caspase-3 induction, OPJ enables neurons to withstand transient oxidative insults that would otherwise be lethal. These findings emphasize the potential of OPJ as a neuroprotective agent and provide a foundation for further studies exploring its mechanisms and therapeutic applications in oxidative stress-related neurodegenerative diseases.

### 2.4. OPJ Augments Antioxidant Defense via Nrf2-HO-1 Signaling Pathway

To determine whether OPJ enhances endogenous antioxidant defense mechanisms under oxidative stress, Nrf2 signaling and the expression of downstream antioxidant enzymes were examined in H_2_O_2_-exposed HT22 neuronal cells.

**Figure 3:**
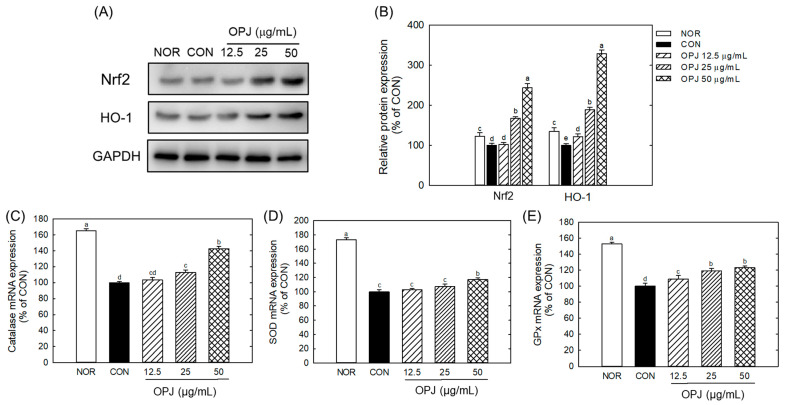
Effect of OPJ on antioxidant response in hydrogen peroxide-exposed HT22 cells. Nrf2 and HO-1 protein abundances were determined by Western blot and their relative levels were quantified by Image J (Version 1.53t) (**A**,**B**). mRNA expressions of catalase (**C**), superoxide dismutase 1 (SOD1) (**D**), and glutathione peroxidase (Gpx) (**E**) were analyzed by real-time PCR. All values represent mean ± SEM. Different letters indicate statistical significance among groups (*p* < 0.05). NOR, normal (untreated) group; CON, H_2_O_2_ (800 μM) treated group.

**Figure 4:**
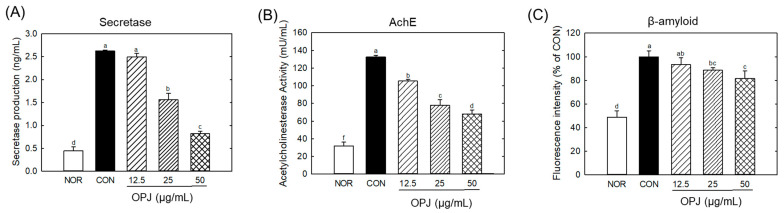
Effect of OPJ on neurogenerative biomarkers in hydrogen peroxide-exposed HT22 cells. β-secretase production (**A**) and acetylcholineesterase (AChE) activity (**B**) were determined by ELISA kit. β-amyloid fibril level (**C**) was examined by Thioflavin staining. All values represent mean ± SEM. Different letters indicate statistical significance among groups (*p* < 0.05). NOR, normal (untreated) group; CON, H_2_O_2_ (800 μM) treated group.

OPJ effectively enhanced the abundance of nuclear factor erythroid 2–related factor 2 (Nrf2), a key regulator of antioxidant responses, and its downstream target, HO-1, under H_2_O_2_-induced oxidative stress conditions (Figure 3A). Nrf2 protein levels increased by 1.6-fold and 2.4-fold at OPJ concentrations of 25 and 50 μg/mL, respectively, while HO-1 levels rose by 1.9-fold and 3.2-fold at the same concentrations (Figure 3B). Furthermore, oxidative stress caused by H_2_O_2_ significantly downregulated the mRNA levels of antioxidant enzymes, including catalase, superoxide dismutase 1 (SOD1), and glutathione peroxidase (Gpx). OPJ treatment counteracted this effect by upregulating the expression of these genes. Catalase mRNA expression increased by 12.5% and 42.4% at 25 and 50 μg/mL of OPJ, respectively (Figure 3C). Similarly, SOD1 mRNA levels showed a modest increase of 10.7% at the lower concentration (not statistically significant) and a significant increase of 17.1% at the higher concentration, while Gpx mRNA levels increased by 18.9% and 23.1% at the corresponding concentrations (Figure 3D,E). These findings suggest that OPJ mitigates oxidative stress-induced neuronal cell death by upregulating the Nrf2 signaling pathway and enhancing the expression of antioxidant enzymes. Nrf2 is a transcription factor that regulates a wide range of antioxidant and cytoprotective genes [21]. Under basal conditions, Nrf2 is bound by Kelch-like ECH-associated protein 1 (KEAP1), which facilitates its ubiquitination and subsequent degradation [24]. Oxidative stress induces conformational changes in KEAP1, releasing Nrf2 to translocate into the nucleus. There, Nrf2 binds to antioxidant response elements (AREs) and induces the expression of enzymes such as superoxide dismutase (SOD), glutathione peroxidase (GPx), and heme oxygenase-1 (HO-1), as observed in this study (Figure 4), confirming the role of Nrf2 in OPJ-mediated antioxidant defense. In addition to its antioxidant role, Nrf2 modulates neuroinflammatory responses by limiting pro-inflammatory cytokines and suppressing the activation of microglia and astrocytes [25]. Moreover, Nrf2 supports neuronal survival by facilitating repair of damaged DNA and proteins [26]. This dual function of Nrf2—attenuating oxidative stress and suppressing neuroinflammation—highlights its significance as a neuroprotective target and provides mechanistic insight into OPJ’s protective effects. Several natural compounds have been identified as Nrf2 activators with therapeutic potential in neurodegenerative diseases. For instance, sulforaphane, found in cruciferous vegetables such as broccoli, has demonstrated neuroprotective effects through Nrf2 activation [27]. Similarly, olive-derived polyphenols have been reported to exert neuroprotective actions via Nrf2 signaling [28]. These protective effects of OPJ are consistent with previous studies on natural Nrf2 activators, suggesting that OPJ may confer neuroprotection by simultaneously enhancing antioxidant defenses and limiting neuroinflammation, reinforcing its potential as a functional food-derived neuroprotective agent.

### 2.5. OPJ Modulates Neurodegenerative Pathological Markers

To assess whether OPJ influences molecular markers associated with early neurodegenerative pathology, β-secretase activity, acetylcholinesterase (AChE) activity, and amyloid fibril formation were evaluated in H_2_O_2_-exposed HT22 neuronal cells.

The level of β-secretase, an enzyme involved in amyloid β production, was significantly elevated under H_2_O_2_-induced oxidative stress. OPJ effectively suppressed β-secretase production, reducing its levels by 40.4% and 68.7% at 25 and 50 μg/mL, respectively (Figure 4A). A similar regulatory effect was observed for acetylcholinesterase (AChE), an enzyme responsible for breaking down acetylcholine, a critical neurotransmitter. OPJ significantly decreased the H_2_O_2_-induced increase in AChE activity by 20.8%, 41.3%, and 48.6% at concentrations of 12.5, 25, and 50 μg/mL, respectively (Figure 4B). Furthermore, OPJ reduced the formation of amyloid fibrils, as detected by thioflavin fluorescence. The fluorescence intensity, indicative of amyloid fibril accumulation, decreased by 11.9% and 19.2% at 25 and 50 μg/mL of OPJ, respectively (Figure 4C). These findings collectively indicate that OPJ effectively suppresses neurodegenerative responses induced by H_2_O_2_-mediated oxidative stress in neuronal cells.

Prolonged oxidative stress is a key driver of early neurodegenerative changes associated with Alzheimer’s disease (AD). In particular, markers such as Tau phosphorylation, β-secretase (BACE1) upregulation, and AChE dysregulation are closely linked to amyloid-β production, cholinergic dysfunction, and cytoskeletal instability, respectively [29,30]. In the present study, H_2_O_2_ exposure markedly increased BACE1, AChE activity, and amyloid fibril accumulation in HT22 cells, reflecting key molecular events of AD-like pathology. OPJ treatment effectively attenuated these H_2_O_2_-induced neurodegenerative markers, indicating a neuroprotective effect. The observed reduction in BACE1 and AChE indicates that OPJ mitigates ROS-mediated molecular alterations that drive early AD pathology. Additionally, the decrease in amyloid fibril formation implies that OPJ can prevent protein aggregation under oxidative stress. These effects are consistent with previous studies showing that polyphenol-rich natural products, such as resveratrol, curcumin and hydroxytyrosol, protect neuronal cells by modulating BACE1 expression and AChE activity in oxidative stress models [31,32]. These findings suggest that OPJ confers multi-level neuroprotection: it preserves neuronal survival, modulates apoptotic pathways, and suppresses early molecular events associated with neurodegeneration. By concurrently targeting ROS accumulation, apoptosis, and key neurodegenerative biomarkers, OPJ demonstrates a protective effect against oxidative stress-induced neuronal injury, highlighting its potential as a functional food-derived intervention for neurodegenerative disorders.

### 2.6. OPJ Enhances Neuronal Resilience and Survival by Activation of AMPKα-SIRT1-PGC1α and CREB-BDNF-TrkB Signaling Pathways

To investigate whether OPJ-mediated neuroprotection is associated with regulation of metabolic and neurotrophic signaling, the AMPKα–SIRT1–PGC1α and CREB–BDNF–TrkB pathways were analyzed in H_2_O_2_-exposed HT22 neuronal cells.

**Figure 5:**
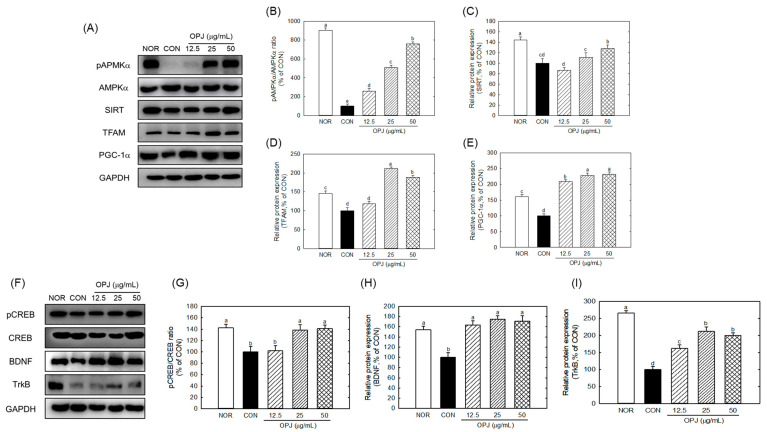
Effect of OPJ on AMPKa-SIRT1-PGC1a and CREB-BDNF-TrkB signaling pathways in hydrogen peroxide-exposed HT22 cells. The components of signaling pathways were determined by Western blot (**A**,**F**). p-AMPKα/total AMPKα (**B**), SIRT1 (**C**), TFAM (**D**), PGC1α (**E**), p-CREB/total CREB (**G**), BDNF (**H**), and TrkB (**I**) protein levels were quantified by Image J (Version 1.53t). All values represent mean ± SEM. Means with different letter showed a significant difference at *p* < 0.05. NOR, normal untreated group; CON, H_2_O_2_ (800 μM) treated group.

OPJ significantly counteracted H_2_O_2_-induced downregulation of AMPKα-SIRT1-PGC1α signaling, which is associated with energy biogenesis and antioxidant responses. OPJ increased phosphorylated AMPKα (p-AMPKα) by 2.5-fold, 5.0-fold, and 7.6-fold at 12.5, 25, and 50 μg/mL, respectively, compared to the H_2_O_2_-treated control (Figure 5A,B). This was accompanied by increased expression of other proteins within the same signaling axis, including SIRT1, PGC1α, and TFAM, as shown in the corresponding Western blot and quantitative analyses (Figure 5A–E). SIRT1 abundance was also elevated with OPJ treatment, increasing by 10.8% and 28.9% at 25 and 50 μg/mL, respectively (Figure 5C). OPJ enhanced PGC1α and its downstream target, TFAM, by 2.0-fold to 2.3-fold at 12.5 to 50 μg/mL and 1.8-fold to 2.1-fold at 25 to 50 μg/mL, respectively (Figure 5D,E). These results indicate that OPJ activates the AMPKα-SIRT1-PGC1α pathway, contributing to neuronal protection against oxidative stress. Additionally, OPJ increased activation of the CREB–BDNF–TrkB signaling pathway. As shown in Figure 5F, OPJ markedly increased CREB phosphorylation under H_2_O_2_-induced oxidative stress conditions in HT22 neuronal cells. This change was accompanied by increased expression of the neurotrophic factor BDNF (Figure 5G,H) and its receptor TrkB (Figure 5I), which are commonly associated with neuronal survival and synaptic function. Consistent with these observations, OPJ increased phospho-CREB by 38.6% and 40.8% at 25 and 50 μg/mL, respectively, while upregulating BDNF protein levels by 63.6% to 75.8% at 25 to 50 μg/mL (Figure 5G,H). TrkB protein levels were also enhanced by 1.6-fold to 2.1-fold at 12.5 to 50 μg/mL (Figure 5I).

Collectively, these findings demonstrate that OPJ is associated with protection of neuronal cells from oxidative stress-induced damage, accompanied by modulation of both AMPKα-SIRT1-PGC1α and CREB-BDNF-TrkB signaling pathways. Figure 5 integrates the concurrent modulation of metabolic (AMPKα–SIRT1–PGC1α) and neurotrophic (CREB–BDNF–TrkB) signaling pathways, providing associative mechanistic insight for the neuroprotective effects of OPJ under oxidative stress conditions.

The simultaneous modulation of metabolic stability (AMPK) and neurotrophic signaling (CREB) represents a notable observation, as OPJ is associated with changes in multiple signaling pathways relevant to oxidative stress and neuronal resilience. AMPK functions as a cellular energy sensor, enhancing mitochondrial function and antioxidant defenses through downstream effectors such as SIRT1 and PGC1α [33]. SIRT1 stabilizes Nrf2 by deacetylation, increasing its DNA-binding affinity, while PGC1α contributes to transcriptional regulation of antioxidant genes [34].

The concurrent upregulation of Nrf2, HO-1, and AMPKα–SIRT1–PGC1α components suggests that OPJ enhances antioxidant defenses via both direct oxidative stress signaling and upstream metabolics, linking energy homeostasis with neuroprotection. This restoration of the AMPK axis highlights a potential therapeutic advantage of OPJ: its ability to address energetic failure, which is a critical upstream deficit in neurodegeneration, distinguishing it from agents that only act as downstream antioxidant scavengers. Beyond its role in regulating oxidative stress, OPJ appears to enhance neuronal resilience by activating the CREB–BDNF–TrkB signaling pathway. CREB (cAMP response element-binding protein) is a transcription factor that promotes the expression of brain-derived neurotrophic factor (BDNF), which in turn binds to its receptor TrkB to support synaptic plasticity, neurogenesis, and neuronal survival [35]. In this study, OPJ treatment enhanced CREB phosphorylation and upregulated both BDNF and TrkB in H_2_O_2_-stressed HT22 cells, consistent with suppressed apoptosis observed in earlier results (Figure 1) [36]. Together, these findings indicate that OPJ exerts neuroprotective effects through the parallel modulation of antioxidative (Nrf2–HO-1), metabolic (AMPKα–SIRT1–PGC1α), and neurotrophic (CREB–BDNF–TrkB) pathways. In this study, modulation of oxidative stress is framed as the primary mechanistic axis, while changes in metabolic, neurotrophic, and inflammatory signaling pathways are interpreted as associated or supportive responses rather than independent primary mechanisms. Such multi-pathway modulation has been infrequently reported for a single natural product, suggesting that OPJ may influence several stress-responsive mechanisms in neuronal cells. Such modulation further supports the therapeutic potential of OPJ as a functional food-derived intervention against neurodegeneration.

### 2.7. OPJ Suppresses Neuroinflammation in BV2 Microglia by Inhibiting NF-κB and MAPK Signaling Pathways

**Figure 6:**
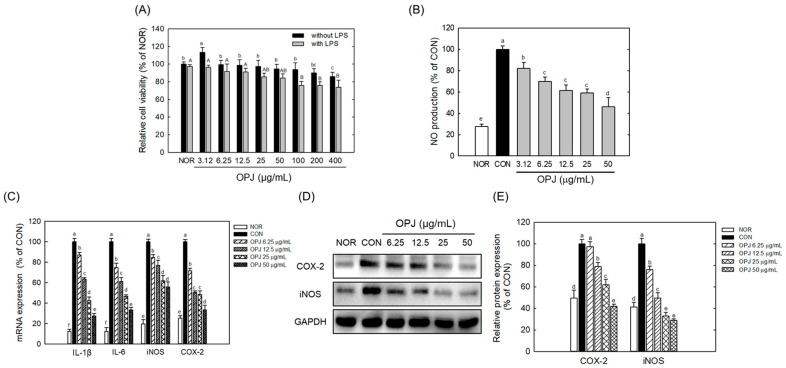
Effect of OPJ on cell viability and inflammatory responses in BV2 cells. BV2 cells were incubated with OPJ (3.125, 6.25, 12.5, 25, 50, 100, 200, 400 μg/mL) with or without lipopolysaccharide (LPS, 100 ng/mL) for 24 h, and then cell viability was measure by MTT assay (**A**). NO generation was determined using Griess reagent (**B**). BV2 cells were treated with OPJ (6.25, 12.5, 25, 50 μg/mL) 2 h pre-treatment before LPS stimulation and incubated for 24 h. mRNA levels of inducible NO (iNOS), COX-2, IL-6, and IL-1β was analyzed by real-time PCR (**C**). Protein abundance of iNOS and COX-2 was determined using Western blot and their levels were quantified using by Image J (Version 1.53t) (**D**,**E**). All values represent mean ± SEM. Means with different letter showed a significant difference at *p* < 0.05. NOR, normal untreated group; CON, only LPS (100 ng/mL) treated group.

To evaluate the anti-inflammatory effects of OPJ under neuroinflammatory conditions, BV2 microglial cells were stimulated with lipopolysaccharide (LPS) and inflammatory mediator production and NF-κB/MAPK signaling were subsequently analyzed. Normal untreated group without LPS was defined as NOR, while LPS-treated cells served as the inflammatory control group (CON). OPJ did not exhibit cytotoxicity in BV2 cells at concentrations ranging from 3.12 to 200 μg/mL in the absence of LPS, whereas a modest reduction in cell viability (approximately 15%) was observed only at 400 μg/mL. In the presence of LPS, OPJ was non-toxic to BV2 cells up to 50 μg/mL, whereas higher concentrations (100–400 μg/mL) resulted in a concentration-dependent reduction in cell viability of approximately 20–30% compared to the NOR group (Figure 6A). OPJ effectively decreased LPS-induced nitric oxide (NO) production, with a 54% reduction in 50 μg/mL (Figure 6B).

OPJ inhibited the inflammatory response in LPS-stimulated BV2 cells. At concentrations of 12.5, 25, and 50 μg/mL, OPJ significantly downregulated the mRNA expression of key inflammatory mediators, including inducible nitric oxide synthase (iNOS) and cyclooxygenase-2 (COX-2), by approximately 20–60% in LPS-stimulated BV2 cells (Figure 6C). Similarly, mRNA levels of the pro-inflammatory cytokines IL-6 and IL-1β, which were elevated under LPS treatment, were reduced by 38.9%, 43.4%, and 66.8%, and 36.3%, 57.4%, and 72.7%, respectively, following OPJ treatment (Figure 6C). In addition to its effects on mRNA expression, OPJ also decreased the protein abundance of iNOS and COX-2. COX-2 protein levels were reduced by 21.1%, 38.0%, and 58.1%, while iNOS protein levels decreased by 50.4%, 67.3%, and 71.3% at 12.5, 25, and 50 μg/mL, respectively (Figure 6D,E). At the lowest concentration tested (6.25 μg/mL), OPJ exhibited detectable but weaker effects on inflammatory marker expression compared with the more pronounced responses observed at concentrations of 12.5 μg/mL and above. These findings indicate that OPJ effectively suppresses the LPS-induced inflammatory response by downregulating both the gene expression and protein abundance of key inflammatory markers.

**Figure 7:**
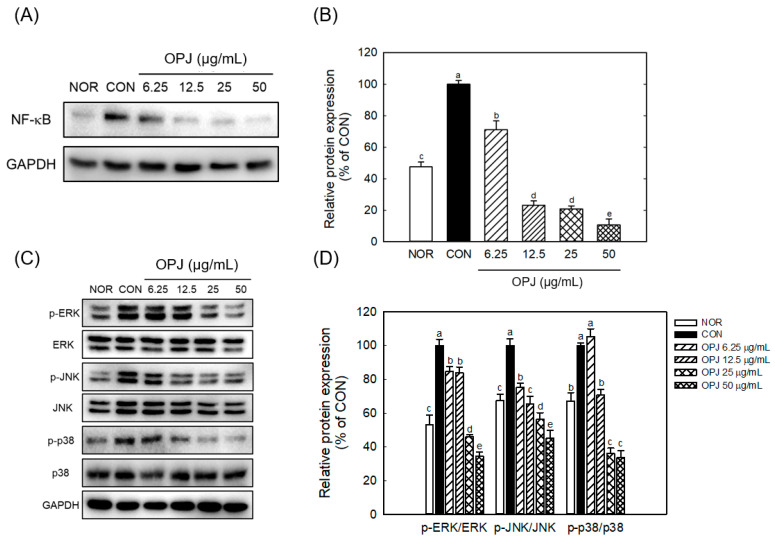
Effect of OPJ on NF-κB and MAPK signaling pathways in LPS-stimulated BV2 cells. NF-κB Protein levels (**A**), and the phosphorylation ratios of ERK, JNK, and p38 were analyzed using Western blot (**A**,**C**) and their levels were quantified using Image J (Version 1.53t) (**B**,**D**). All values represent mean ± SEM. Means with different letter showed a significant difference at *p* < 0.05. NOR, normal untreated group; CON, only LPS treated group (100 ng/mL).

OPJ further suppressed key inflammatory signaling pathways, including nuclear factor kappa B (NF-κB) and mitogen-activated protein kinases (MAPKs). The LPS-induced increase in total NF-κB protein levels was significantly reduced by OPJ treatment, with decreases of 76.9%, 79.2%, and 89.4% at concentrations of 12.5, 25, and 50 μg/mL, respectively (Figure 7A,B). Additionally, OPJ reduced the phosphorylated levels of three major components of the MAPK pathway: extracellular signal-regulated kinase (ERK), c-Jun N-terminal kinase (JNK), and p38 (Figure 7C). Specifically, phosphorylated ERK levels were reduced by 16.2%, 54.1%, and 65.5%; phosphorylated JNK levels decreased by 34.6%, 43.8%, and 54.7%; and phosphorylated p38 levels were lowered by 29.5%, 64.0%, and 66.4% at 12.5, 25, and 50 μg/mL, respectively, compared to the control group (Figure 7D).

These findings demonstrate that OPJ is associated with suppression of inflammatory responses, accompanied by reduced NF-κB protein abundance and decreased phosphorylation of MAPK signaling components. The anti-inflammatory effects of OPJ are founded on its ability to directly suppress the central signaling pathways of inflammation (Figure 7). NF-κB and MAPK family (ERK, JNK, p38) play critical, interconnected roles in cellular stress responses and neuroinflammation. NF-κB is a master transcription factor regulating the expression of inflammatory mediators, which is often hyperactivated by oxidative stress and cytokines in neurodegenerative conditions, leading to chronic inflammation that directly harms neurons. Our findings demonstrated that OPJ effectively counteracted this inflammatory cascade. The reduction in inflammatory output (NO, iNOS, COX-2, and cytokines) is associated with decreased NF-κB protein levels and concurrent attenuation of MAPK pathway activation (Figure 7). Specifically, OPJ markedly reduced the phosphorylation of all three MAPK components (ERK, JNK, p38). The attenuation of p38 and JNK signaling is considered important, as these pathways are closely associated with pro-inflammatory and pro-apoptotic signaling processes involved in neurodegeneration, including JNK’s role in neuronal apoptosis and p38’s role in cytokine production [37,38,39]. Critically, the suppression of ERK (up to 65.5% reduction) is also highly relevant. Although ERK typically supports neuronal survival, its chronic over-activation is linked to pathological outcomes such as Tau hyperphosphorylation and neurofibrillary tangle formation [38]. Therefore, OPJ-mediated modulation of MAPK family members is consistent with attenuation of signaling processes involved in oxidative stress and neuroinflammation [37,38,39]. Furthermore, this mechanism complements OPJ’s other neuroprotective effects: Nrf2, a key antioxidative factor activated by OPJ, is known to mitigate oxidative stress and reduce the upstream triggers of both NF-κB and MAPKs signalings [40]. Overall, the observed increase in Nrf2-related responses, together with modulation of NF-κB protein levels and MAPK phosphorylation, is consistent with reduced oxidative and inflammatory signaling, supporting the potential relevance of OPJ as a functional food-derived bioactive.

### 2.8. Comparative Biological Effects of Hydroxytyrosol and Tyrosol in Relation to OPJ-Mediated Neuroprotection

**Figure 8:**
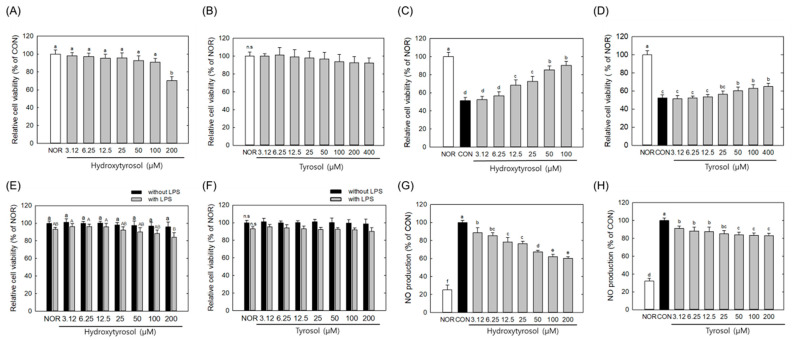
Comparative effects of hydroxytyrosol and tyrosol on cell viability and inflammatory response in HT22 and BV2 cells. HT22 cells were incubated with hydroxytyrosol or tyrosol (3.125 to 400 μM) (**A**,**B**), in the absence and presence of H_2_O_2_ (**C**,**D**) for 24 h, and then cell viability was measure by MTT assay. BV2 cells were incubated with hydroxytyrosol or tyrosol (3.125 to 200 μM) with or without lipopolysaccharide (LPS) for 24 h, and then cell viability was measure by MTT assay (**E**,**F**). BV2 cells were treated with hydroxytyrosol or tyrosol (6.25 to 200 μg/mL) 2 h before LPS stimulation and incubated for 24 h. NO generation was determined using Griess reagent (**G**,**H**). All values represent mean ± SEM. Means with different letter showed a significant difference at *p* < 0.05. NOR, normal untreated group; CON, H_2_O_2_-treated group (HT22 cells) or LPS-treated group (BV2 cells). n.s indicates no statistically significant difference.

To compare the relative cellular activities of the major OPJ-derived phenolic compounds, hydroxytyrosol and tyrosol were assessed under equivalent experimental conditions for their effects on oxidative stress-induced neuronal viability in HT22 cells and on LPS-induced inflammatory responses in BV2 microglial cells. It should be noted that hydroxytyrosol and tyrosol were tested at equivalent concentrations to enable direct comparison of their intrinsic cellular activities, rather than to replicate their exact relative abundance within OPJ. Therefore, these results should be interpreted as comparative activity profiles rather than quantitative contributions within the extract. Hydroxytyrosol and tyrosol were non-toxic at concentrations of 100 μM and 400 μM, respectively, in HT22 cells (Figure 8A,B). Both compounds enhanced neuronal cell viability under H_2_O_2_-induced oxidative stress conditions, increasing cell viability by 76.1% and 24.2%, respectively, at a concentration of 100 μg/mL compared to the CON group (Figure 8C,D). This indicates that hydroxytyrosol exhibits greater protective activity than tyrosol under these conditions, while the magnitude and breadth of the OPJ-mediated neuroprotective effects observed in earlier sections suggest additional contributions from other OPJ constituents beyond individual phenolic compounds. In BV2 cell viability assays, both compounds showed non-toxic effects at concentrations up to ~200 μM (Figure 8E,F). Hydroxytyrosol and tyrosol decreased NO production by 40.0% and 17.6%, respectively, at a 100 μM concentration (Figure 8G,H), suggesting that hydroxytyrosol suppresses the LPS-induced inflammatory response more effectively. While hydroxytyrosol is identified as the more bioactive compound in these comparative assays, OPJ contains tyrosol together with a substantial proportion of other unidentified polyphenols (21.46%), which may act cooperatively to shape the overall biological responses observed at the extract level. This suggests that the comprehensive protective effects of OPJ might be due to a ‘cocktail effect’ rather than the action of a single compound alone. Consistent with this interpretation, previous studies have reported that olive-derived phenolics, including hydroxytyrosol and oleuropein, modulate amyloid aggregation and neuronal survival, supporting the view that individual phenolic activities may partially explain, but do not fully account for, the broader biological effects of olive-based extracts [41,42]. Moreover, a recent study introduced a hybrid molecule combining hydroxytyrosol with donepezil, a well-established Alzheimer’s drug, underscoring its therapeutic potential [43]. In addition to hydroxytyrosol and oleuropein, oleocanthal has also been reported as a potent olive-derived phenolic compound with strong biological effects at the neuronal level, including modulation of neuroinflammatory signaling and attenuation of amyloid-related neurotoxicity [44,45]. In the context of the present study, these observations support the interpretation that hydroxytyrosol contributes to, but does not solely define, the neuroprotective and anti-inflammatory effects of OPJ. Although phenolic compounds such as oleuropein are often considered key antioxidant and anti-inflammatory agents in olive-based products, our HPLC analysis did not detect oleuropein.

Because OPJ-derived compounds must access the central nervous system to exert direct neuroprotective effects, blood–brain barrier (BBB) permeability is a relevant consideration. Hydroxytyrosol, a small (154.16 Da), lipophilic molecule, is reported to readily penetrate the BBB, whereas tyrosol exhibits comparable permeability but lower bioactivity, consistent with its weaker cellular effects observed in this study [46,47]. While other unidentified phenolic compounds in OPJ may contribute to neuroprotection, our findings indicate that hydroxytyrosol is the predominant phenolic compound in OPJ and a major contributor to its observed neuroprotective and anti-inflammatory effects. Although hydroxytyrosol was included in comparative cellular assays as a reference compound, the majority of mechanistic experiments in this study were conducted using the OPJ extract. Therefore, the observed neuroprotective and anti-inflammatory effects should be interpreted as extract-level phenomena rather than as effects attributable to a single compound. The potential contributions of tyrosol and other unidentified polyphenols, as well as synergistic interactions among OPJ constituents, should also be considered.

#### Limitations

The present study has several limitations that should be acknowledged. First, all findings were derived from in vitro models of oxidative stress-induced neuronal damage (HT22 cells) and LPS-stimulated microglial activation (BV2 cells), which do not fully recapitulate the complexity of neurodegenerative diseases in vivo. Second, although hydroxytyrosol has been reported to cross the blood–brain barrier, its oral bioavailability is limited, as it is rapidly metabolized into glucuronide and sulfate conjugates before reaching the systemic circulation and the brain. The neuroprotective efficacy and BBB permeability of these OPJ-derived metabolites may differ substantially from those of the parent compound tested in this in vitro study. Finally, the concentrations used in vitro were selected to investigate relative cellular responses and mechanistic associations, and therefore do not directly reflect achievable brain concentrations in vivo. Consequently, the present findings should be interpreted as providing mechanistic insight into oxidative stress– and inflammation-associated neurodegenerative processes, rather than as evidence of disease prevention or therapeutic efficacy, highlighting the need for future in vivo and pharmacokinetic studies.

## 3. Materials and Methods

To investigate the neuroprotective and anti-inflammatory potential of olive pomace juice (OPJ), an in vitro experimental framework was established using two complementary cell models. Mouse hippocampal neuronal HT22 cells were employed to evaluate oxidative stress-induced neuronal injury and associated signaling pathways, whereas BV2 microglial cells were used to assess lipopolysaccharide (LPS)-induced neuroinflammatory responses. OPJ was selected as the primary test material to reflect extract-level biological effects, while hydroxytyrosol and tyrosol were included as reference compounds for comparative analysis of major OPJ-derived phenolics. Oxidative stress and inflammatory conditions were induced using hydrogen peroxide (H_2_O_2_) and LPS, respectively, based on preliminary optimization experiments. The overall experimental sequence comprised OPJ compositional analysis, determination of non-cytotoxic concentration ranges, induction of oxidative stress or inflammation, and subsequent evaluation of cellular viability, signaling pathways, and functional biomarkers.

A schematic overview of the experimental workflow is provided in Figure 1. Based on this design, the Results and Discussion are organized to first describe OPJ composition, followed by its neuroprotective effects in HT22 neuronal cells and anti-inflammatory effects in BV2 microglial cells.

### 3.1. Preparation of OPJ

Olive pomace juice (OPJ) used in this study was obtained from JYGlobalfoods Co., Ltd. (Seoul, Republic of Korea) and was prepared through a three-step process. First, ripe olive fruits (*Olea europaea* L.) were washed with water to remove impurities before being milled to facilitate oil extraction. The resulting mixture then underwent horizontal centrifugation, which separated it into two primary components: olive oil, the main product for consumption, and alpeorujo, a byproduct containing residual oil, water, and solids. In the second step, the alpeorujo was further processed through centrifugation, yielding three key components: solids, pomace oil, and olive vegetation water (also known as olive pomace juice). This olive vegetation water was then subjected to filtration and concentration to refine its composition. In the final step, the filtered and concentrated olive vegetation water underwent further processing through an exchange column process using osmosis water for enhanced purification. The purified liquid was then subjected to evaporation for heat sterilization. Following this, a non-GMO dextrin (as a carrier) and a small amount of calcium salts (as an anticaking agent) were added before the mixture entered a vacuum drying stage. The dried material was then milled into a fine powder (OPJ). OPJ was standardized to contain more than 10.0% hydroxytyrosol and 2.0% tyrosol (*w*/*w*) based on HPLC analysis (see Section 3.9) across three independent batches.

### 3.2. MTT Assay

HT22 or BV2 cells (1.0 × 10^5^ cells/mL, a gift from Korea University) were cultured in Dulbecco’s Modified Eagle’s Medium (DMEM) containing 10% fetal bovine serum and 1% penicillin-streptomycin. When the cell density reached 70%, the cells were subcultured to maintain the culture. The cells were seeded in a 96-well plate and incubated for 24 h. Afterward, OPJ with indicated concentrations was introduced to the cells, and the culture was maintained for 24 h. After 24 h, the medium was replaced with a serum-free medium containing 3-(4,5-dimethylthiazol-2-yl)-2,5-diphenyltetrazolium bromide (MTT) reagent (0.5 mg/mL, Sigma-Aldrich, St. Louis, MO, USA), and the cells were incubated at 37 °C for 1 h. The medium was then removed, and dimethyl sulfoxide (DMSO) was added to each well to dissolve the MTT formazan. The absorbance was measured at 550 nm using a microplate reader (Spectra Max M3, Molecular Devices, San Jose, CA, USA). Untreated cells without OPJ, H_2_O_2_, or LPS exposure were used as the normal control (NOR), whereas cells treated with H_2_O_2_ alone (HT22 cells) or LPS alone (BV2 cells) served as the respective experimental control (CON). Culture medium without cells was used as the blank for background subtraction.

### 3.3. Cell Culture

The HT22 or BV2 cells (1.0 × 10^5^ cells/mL) were initially seeded in a 6-well plate and cultured for 24 h in Dulbecco’s Modified Eagle’s Medium (DMEM, Welgene, Gyeongsan-si, Republic of Korea) containing 3.7 g/L sodium bicarbonate, 10% fetal bovine serum (FBS, Welgene, Gyeongsan-si, Republic of Korea), and 1% penicillin-streptomycin (PS, Welgene, Gyeongsan-si, Republic of Korea). The cells were then incubated at 37 °C with 5% CO_2_ for an additional 24 h. To investigate the influence of OPJ on H_2_O_2_-induced cell death, cells were treated with OPJ for 2 h. Subsequently, H_2_O_2_ (800 μM, St. Louis, MO, USA) was administered to induce oxidative stress. To evaluate the effect of OPJ on the inflammatory response, cells were pretreated with OPJ for 2 h before stimulation with LPS (100 ng/mL) and incubated for 24 h. Cells cultured without H_2_O_2_ or LPS treatment were used as the normal control (NOR), while cells exposed to H_2_O_2_ (800 μM) or LPS (100 ng/mL) alone were used as the oxidative stress or inflammatory control (CON), respectively.

### 3.4. Determination of NO Production

Cells were seeded in 6-well plates at a concentration between 1 × 10^5^ and 2 × 10^5^ cells/well and cultured for 24 h. Afterward, OPJ is treated for 2 h, followed by additional 24 h of incubation with LPS. Then, cell culture medium (100 μL) and Griess reagent (100 μL) were added to a 96-well plate and allowed to react at 25 °C for 5 min. The amount of nitric oxide (NO) is then determined by measuring the absorbance at 550 nm using a microplate reader (INNO M, LTek, Seongnam, Republic of Korea). Cells treated with culture medium alone were used as the normal control (NOR), and cells treated with LPS alone were used as the inflammatory control (CON). Culture medium without cells was used as the blank for NO measurement.

### 3.5. Determination of Intracellular Amyloid Fibrils

Thioflavin (Sigma-Aldrich, St. Louis, MO, USA), a cell-permeable benzothiazole dye, was prepared in PBS buffer at a concentration of 10 μM. After removing the culture medium, the dye was applied to the cells and incubated at 37 °C for 2 h in the dark. Fluorescence was then measured using a SpectraMax M3 fluorometer (Molecular Devices, Sunnyvale, VA, USA) with an excitation wavelength of 437 nm and an emission wavelength of 485 nm. Untreated cells were used as the normal control (NOR), whereas cells treated with H_2_O_2_ alone served as the oxidative stress control (CON).

### 3.6. Western Blot

After treating the cells with the sample and incubating for 24 h, the medium was removed. Lysis buffer containing protease inhibitors (aprotinin, leupeptin, phenylmethylsulfonyl fluoride [PMSF], Thermo Fisher Scientific Inc., Waltham, MA, USA) and phosphatase inhibitors was added to lyse the cells. The lysed cells were collected and centrifuged at 10,000× *g* for 10 min at 4 °C. The supernatant was collected to obtain the protein extract. Using the Bicinchoninic Acid (BCA) assay, an amount of the extract corresponding to 50 μg of protein was separated by electrophoresis on a gel containing 10% acrylamide (GeneAll Biotechnology Co., Ltd., Seoul, Republic of Korea). The proteins on the gel were transferred onto a polyvinylidene fluoride (PVDF) membrane. The membrane was blocked with 5% skim milk for 30 min, followed by incubation with a primary antibody at a 1:1000 dilution at 4 °C for 24 h. The membrane was washed twice with Tris-buffered saline with 0.1% Tween 20 (TBST) for 10 min each. It was then incubated with a secondary antibody at 25 °C for 2 h. After additional washes with TBST, the target proteins on the membrane were visualized using enhanced chemiluminescence (ECL) reagents. Protein bands were detected using the Alliance Q9 system (UVITECH, Cambridge, UK) and analyzed with Alliance Q9 software. The relative intensity of protein bands was quantified using ImageJ software. Primary antibodies used in this study are follows: BAX, BCL-2, procaspase-3, cleaved caspase-3, p-ERK, ERK, p-JNK, JNK, p-p38, p38, COX-2, iNOS, NF-κB, phospho-AMPKα, total AMPKα, SIRT1, PGC1α, phospho-CREB, total CREB, BDNF, and TrkB were purchased from Cell Signaling Technology, Inc (Denvers, MA, USA). Nrf2, heme oxygenase-1 (HO-1), and glyceraldehyde-3-phosphate dehydrogenase (GAPDH) were obtained from Santa Cruz Biotechnology, Inc. (Dallas, TX, USA). The secondary antibody (anti-rabbit IgG, horseradish peroxidase conjugated) was obtained from Cell Signaling Technology, Inc (Danvers, MA, USA). Protein expression levels were compared relative to untreated cells (NOR) or cells treated with H_2_O_2_ or LPS alone (CON), depending on the experimental condition.

### 3.7. RNA Extraction and Real-Time PCR

Total RNA was extracted from collected cells using TRIzol reagent (Thermo Fisher Scientific) following the manufacturer’s protocol. After centrifugation at 12,000 rpm for 8 min at 4 °C, the supernatant was mixed with isopropanol, incubated for 1 h, and centrifuged again to obtain an RNA pellet. The pellet was washed with 70% ethanol, air-dried at 25 °C, and dissolved in 30–50 μL of diethyl pyrocarbonate (DEPC)-treated water. cDNA was synthesized from 1 μg of RNA using the Maxime RT PreMix kit (iNtRON Biotechnology, Seongnam, Republic of Korea). qPCR was performed using SYBR Green PCR master mix (Applied Biosystems, Waltham, MA, USA) and the AriaMX real-time PCR system (Agilent Technology, Santa Clara, CA, USA). The primers used are listed in Table 1. Gene expression levels were normalized to GAPDH and compared relative to untreated control cells (NOR) or cells treated with H_2_O_2_ or LPS alone (CON).

### 3.8. Determination of β-Secretase Production and Acetylcholineesterase (AChE) Activity

The activity of β-secretase and acetylcholinesterase (AChE) in HT22 cells was evaluated using the β-secretase activity kit (R&D Systems, Wiesbaden, Germany) and the acetylcholinesterase activity kit (BM-ACH-100, Biomax, Guri, Republic of Korea), respectively, in accordance with the manufacturers’ instructions. Untreated HT22 cells were used as the normal control (NOR), and cells treated with H_2_O_2_ alone served as the oxidative stress control (CON).

### 3.9. HPLC Analysis of Hydroxytyrosol and Tyrosol

Hydroxytyrosol and tyrosol samples were analyzed using an Alliance 1260 HPLC system (Waters, Milford, MA, USA) equipped with an autosampler, quaternary pump, and UV detector. Samples were prepared by dissolving 5 mg of each compound per mL of dimethyl sulfoxide (DMSO) and filtering through a 0.45 μm nylon membrane. The filtered supernatant was injected into a LIChrospher 100-C18 column (250 × 4.0 mm, 5 μm particle size) at 30 °C. Detection was performed at a wavelength of 280 nm. A gradient elution was carried out with a mixture of acetic acid (2.5%) and acetonitrile in the following ratios: 95:5 (at 0 min), 75:25 (20 min), 50:50 (40 min), 20:80 (50 min), and returning to 95:5 (60 min). The flow rate was maintained at 1.0 mL/min with an injection volume of 20 μL. HPLC analyses were performed on three independent batches of OPJ to confirm consistent content of key bioactive compounds. Quantification of hydroxytyrosol and tyrosol in OPJ was carried out by comparing peak areas to those of pure standards. Calibration curves were constructed using serial dilutions of each standard compound at appropriate concentrations. Each analyte showed excellent linearity within its respective range with coefficients of determination (R^2^) exceeding 0.99. As a result, the final OPJ contains 10.92% hydroxytyrosol and 2.18% tyrosol (*w*/*w*). Hydroxytyrosol (4999S) and tyrosol (4949S) standards were obtained from EXTRAXYNTHESE (Genay Cedex, France). In addition to HPLC analysis, the total polyphenol content of OPJ was determined using a colorimetric assay based on the Folin–Ciocalteu method. Total polyphenol content was expressed as gallic acid equivalents (GAE) based on a calibration curve constructed using gallic acid as the standard, and the results were calculated as percentage (*w*/*w*) relative to the dry weight of OPJ.

### 3.10. Statistical Analysis

Each experiment was performed at least three times, and the data were analyzed using one-way analysis of variance (ANOVA) with Statistical Package for the Social Sciences (SPSS; version 27.0; IBM-SPSS Inc., Chicago, IL, USA). Statistical significance among mean values was determined at the *p* < 0.05 level followed by Duncan’s multiple range test. Results are expressed as the mean ± standard error of the mean (SEM). Changes less than 100% relative to control are expressed as percent change, whereas changes equal to or greater than 100% are expressed as fold change relative to control.

## 4. Conclusions

Our findings highlight the potential of olive pomace juice (OPJ) as a functional food-derived material with neuroprotective and anti-inflammatory properties under conditions of oxidative stress and neuroinflammation. OPJ treatment was associated with improved neuronal survival, attenuation of apoptosis, enhancement of antioxidant defense, and suppression of inflammatory signaling in HT22 neuronal cells and BV2 microglia. Hydroxytyrosol, identified as a principal bioactive component of OPJ, contributes substantially to the observed cellular protective effects; however, the majority of mechanistic analyses were conducted using the OPJ extract. Accordingly, the observed biological activities should be interpreted as extract-level phenomena, potentially involving synergistic interactions among multiple polyphenolic constituents. Importantly, the present findings are derived exclusively from in vitro models of oxidative stress-induced neuronal damage and LPS-stimulated microglial activation. Therefore, these results should be interpreted as providing mechanistic insight into processes associated with neurodegeneration rather than as evidence for disease prevention or therapeutic efficacy. Further in vivo and pharmacokinetic studies are required to clarify bioavailability, metabolic transformation, and pathway hierarchy underlying OPJ-mediated effects.

Beyond its biological relevance, OPJ—derived from olive pomace, an agro-industrial by-product—represents a promising candidate for upcycling into value-added ingredients for the food and nutraceutical industries, supporting circular economy principles [48,49,50] while offering potential health-related benefits.

## Figures and Tables

**Table 1:** Primers used in this study.

Name	Forward (5′ to 3′)	Reverse (5′ to 3′)
iNOS	CAGCCAAGCACTCCAATGTA	CCTGAAGGAGCTTTGTCCAC
COX2	TCTCCCCTCTCTACGCATTC	GGCAGAACGACTCGGTTATC
IL-1β	ACTCATTGTGGCTGTGGAGA	TTGTTCATCTCGGAGCCTGT
IL-6	CTGCTGCACTTTGGAGTGAT	GCCTCTTTGCTGCTTTCACAC
Gpx	AGGGTAGAGGC CGGATAAG	AGAAGGCATACACGGTGGAC
Catalase	ACATGGTCTGGGACTTCTGG	CAAGTTTTTGATGCCCTGGT
SOD1	GAGACCTGGGCAATGTGACT	GTTTACTGCGCAATCCCAAT
GAPDH	CTGCGACTTCAACAGCAACT	GAGTTGGGATAGGGCCTCTC

## Data Availability

The original contributions presented in the study are included in the article.

## Supplementary Materials

The following supporting information can be downloaded at: https://www.mdpi.com/article/10.3390/molecules31020336/s1. Figure S1. HPLC chromatogram of OPJ and standards. Standards (hydroxytyrosol and tyrosol) (A,B) and OPJ (C) were analyzed using HPLC. The HPLC chromatogram represents the relative distribution of major and minor phenolic constituents and was not used to calculate the total poly-phenol content.

## Abbreviations

AChE, Acetylcholineesterase; AMPK, AMP-activated protein kinase; BACE-1, Beta-site amyloid precursor protein cleaving enzyme-1 (beta-secretase); BAX, BCL associated X; BBB, Blood brain barrier; BCL2, B-cell lymphoma; BDNF, brain-derived neurotrophic factor; COX-2, Cyclooxigenase-2; CREB, Cyclic AMP Response Element Binding Protein; ELISA, Enzyme-Linked Immunosorbent Assay; ERK, Extracellular Signal-Regulated Kinase; Gpx, Glutathione peroxidase; HO-1, Hemoxygenase-1; iNOS, inducible nitricoxide synthase; JNK, c-Jun N-terminal Kinase; Keap1, Kelch-like ECH-associated protein 1; IL-1β/6, Interleukin-1beta; NF-κB, Nuclear Factor kappa-light-chain-enhancer of activated B cells; Nrf2, Nuclear factor erythroid 2-related factor 2; MAPKs, Mitogen-Activated Protein Kinases; OPJ, Olive pomace extract; PGC1a, Peroxisome proliferator-activated receptor gamma coactivator 1-alpha; ROS, Reactive oxygen species; SIRT1, NAD-dependent deacetylase sirtuin-1; SOD1, superoxide dismutase 1; TrkB, tropomyosin receptor kinase B.
