# Supplementary material for: Standardized Hydroxytyrosol-Enriched Olive Pomace Juice Modulates Metabolic and Neurotrophic Signaling Pathways to Attenuate Neuroinflammation and Protect Neuronal Cells
Source: Molecules. 2026 Jan 19;31(2):336. doi: 10.3390/molecules31020336 (PMC12844416; doi:10.3390/molecules31020336)
Supplement: Supplementary file 1 [file molecules-31-00336-s001.zip › molecules-4039222-supplementary.pdf]

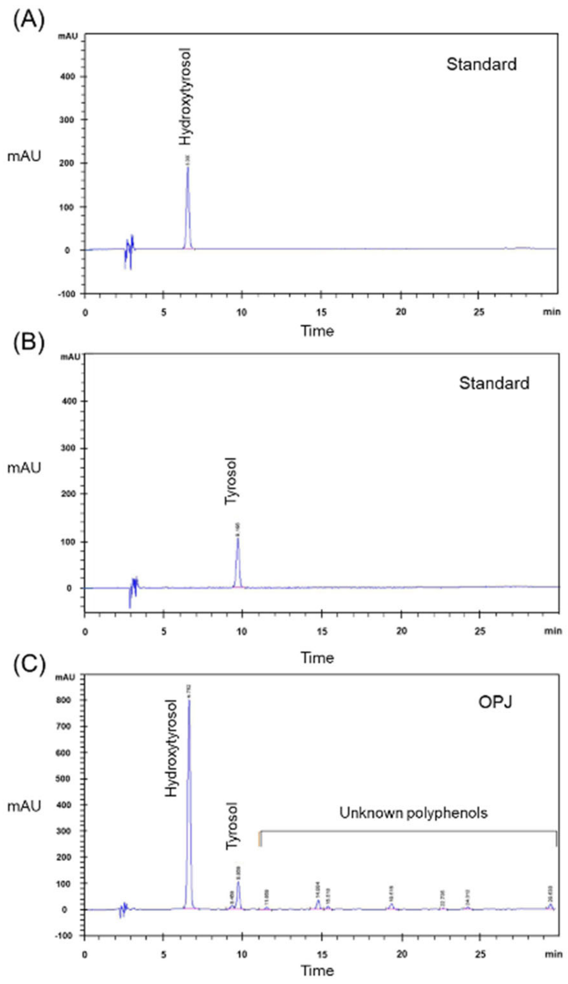

Figure S1. HPLC chromatogram of OPJ and standards. Standards (hydroxytyrosol and tyrosol) (**A**,**B**) and OPJ (**C**) were analyzed using HPLC. The HPLC chromatogram represents the relative distribution of major and minor phenolic constituents and was not used to calculate the total polyphenol content.
